# Auditory working memory for objects vs. features

**DOI:** 10.3389/fnins.2015.00013

**Published:** 2015-02-09

**Authors:** Sabine Joseph, Sukhbinder Kumar, Masud Husain, Timothy D. Griffiths

**Affiliations:** ^1^Institute of Cognitive Neuroscience, University College LondonLondon, UK; ^2^Institute of Neurology, University College LondonLondon, UK; ^3^Wellcome Trust Centre for Neuroimaging, University College LondonLondon, UK; ^4^Institute of Neuroscience, Medical School, Newcastle UniversityNewcastle, UK; ^5^Nuffield Department of Clinical Neurosciences, University of OxfordOxford, UK; ^6^Department of Experimental Psychology, University of OxfordOxford, UK

**Keywords:** auditory, working memory, object, feature, representation

## Abstract

This work considers bases for working memory for non-verbal sounds. Specifically we address whether sounds are represented as integrated objects or individual features in auditory working memory and whether the representational format influences WM capacity. The experiments used sounds in which two different stimulus features, spectral passband and temporal amplitude modulation rate, could be combined to produce different auditory objects. Participants had to memorize sequences of auditory objects of variable length (1–4 items). They either maintained sequences of whole objects or sequences of individual features until recall for one of the items was tested. Memory recall was more accurate when the objects had to be maintained as a whole compared to the individual features alone. This is due to interference between features of the same object. Additionally a feature extraction cost was associated with maintenance and recall of individual features, when extracted from bound object representations. An interpretation of our findings is that, at some stage of processing, sounds might be stored as objects in WM with features bound into coherent wholes. The results have implications for feature-integration theory in the context of WM in the auditory system.

## Introduction

The auditory scene contains sound sources, which can be parsed into auditory objects by the listeners. Some investigators have argued that auditory objects might be considered to be fundamental perceptual units, comprising combinations of sensory cues that form a coherent whole that may or may not be associated with a semantic label (Griffiths and Warren, 2004). Characteristics of stimulus structure, such as common onsets and offsets, harmonic structure, and common modulation determine object perception (Bregman, 1990; Darwin and Carlyon, 1995). Auditory objects can be argued to be the unit of auditory perception subject to sequential grouping to form larger “streams” that might be obligatory (Vliegen et al., 1999) or dependent to an extent upon selective attention (Shinn-Cunningham, 2008).

Only a single previous study (Mathias and Von Kriegstein, 2014) explored the possibility that auditory objects might be fundamental units of working memory (WM) as well as perception (Griffiths et al., 2009). In a selective interference paradigm, Mathias and Von Kriegstein (2014) showed that auditory information (distractors) presented in the delay period influenced recall of auditory objects, composed of multiple features. Using a fundamentally different paradigm in current study, we seek to investigate further whether auditory objects or their constituent cues are represented in working memory. If auditory objects are represented in WM as bound units (objects) as opposed to individual features there may be differences in the accuracy of encoding, maintenance and recall of objects, as opposed to features, and interference between individual features in WM. In contrast to the study by Mathias and Von Kriegstein (2014), which showed that information from distractors presented in the delay period influences recall of single auditory objects, we test how different types of information contained within a single object influence each other. Additionally, we test for effects of memory load (single vs. multiple auditory objects) on accuracy of recall as a function of feature or object storage.

In the current study, we examine WM for artificial auditory objects in which we can manipulate two stimulus features independently to produce distinct perceptual attributes of the sound: amplitude modulation (AM) and spectral passband. These two features represent orthogonal manipulations of the frequency-time structure of the acoustic stimulus (although independent manipulation is impossible in mathematical and physical terms). At the low rates used here sinusoidal amplitude modulation produces a change in the timbre of the object. Spectral-passband manipulation can be argued to produce a change in the timbre or pitch height of stimuli (see Sethares, 2005; for discussion of pitch height and timbre). Whether the manipulation affects pitch height or timbre is unimportant here. The critical point is that the AM rate and passband are distinct features of artificial auditory objects and the use of such objects allows us to examine the relative importance of object features vs. objects systematically.

We seek to define how these features are integrated into coherent wholes within working memory. In visual perception, the binding problem refers to how distributed neural codes representing different features of the perceptual scene are recombined so that one perceives the actual object (Treisman and Schmidt, 1982). Distinct but partially overlapping neural populations are posited that code for different features, where any two features compete for “representational space” in the underlying neural regions, resulting in over-writing or partially corrupted representations. We address here whether such a code might exist in auditory working memory: this predicts interference across features or a feature-extraction cost for individual dimensions of sound compared to the auditory objects with which they are associated. Such a finding would support auditory objects rather than the component features as the units of auditory working memory.

In the current study, subjects performed a within-modality dual task. They had to memorize sequences of variable length (1–4 items) composed of auditory objects containing two features (spectral passband and temporal AM rates). Subjects had to maintain in WM the objects as a whole or their individual features until recall for one of the items was tested by means of two-alternative forced choice (2AFC). We would like to emphasize that this is an active task, where (1) stimuli used were difficult to code verbally, which had to be maintained actively to prevent forgetting; (2) having to ignore individual features demands mental manipulation of stimuli. Thus, the task taps in to working memory rather than more passive short-term memory storage.

We measures recall accuracy and scores were compared across conditions and memory loads. We found that memory performance was best when subjects had to hold the *object in mind*, compared to performance on the individual feature conditions. One interpretation of our findings is that at some stage of processing sounds might be stored as objects in WM, as there is an extraction cost when encoding and recollecting single features stored in WM. Additionally, lower accuracy on individual feature conditions may also be due to interference across features within objects. We suggest that a combination of feature extraction and interference costs explain our results best. Our findings support the idea that we naturally remember sounds as bound objects, even when asked to memorize single constituent features only.

## Materials and methods

### Participants

10 healthy young adults (5 female, mean age 24 years, age range: 19–39) participated in the main experiment and initial control experiment. Another 10 participants completed an additional control experiment (5 female, mean age 24 years, age range: 19–39). All participants provided written informed consent to procedures approved by the UCL research ethics committee. Participants were selected based on the following criteria: normal hearing and no musical training, as assessed by self-report on questionnaires.

### Stimuli and apparatus

Stimuli comprised auditory objects with two features. Both features are fundamental components of natural sounds, relevant to the perception of timbre. The first sound feature is the spectral passband of sound, similar to the spectral centroid dimension identified by multidimensional scaling studies (Grey, 1977; Handel, 1995; McAdams et al., 1995; Samson et al., 1997). Centre frequency was selected from a fixed set of 8 values from the range of 250–1500 Hz. There was a minimum separation of 29% between any two succeeding values in the set, resulting in the following set of center frequencies: 250, 322.92, 417.12, 538.8, 695.98, 899, 1161, 1500 Hz. Narrow band noise was created with these spectral frequencies and a total bandwidth of one quarter of an octave. The second sound feature is the temporal AM rate of sound, relevant to temporal dimensions of timbre identified by multidimensional scaling such as attack time. Another fixed set of 8 values was generated from the range of 6–32 Hz with a minimum separation of 28%, resulting in the following set of values: 6, 7.62, 9.67, 12.29, 15.61, 19.83, 25.19, 32 Hz. Sinusoidal amplitude modulation was applied to the narrow band noise with these rates and a depth of 100%. The spacing of cues within each stimulus dimension was log spaced to account for the increase in threshold for AM rate discrimination as a function of AM rate (Lee, 1994) and the increase in threshold for spectral frequency discrimination as a function of frequency (Micheyl et al., 2012). The AM rate increments were all more than four times threshold and the frequency changes all more than 100 times threshold.

A sound attribute of each dimension [spectral (S) and temporal (T)] was selected at random from each set without replacement. Attributes (S and T) were combined to form auditory objects, e.g., object 1 with spectral passband of 538.8 Hz and temporal AM rate of 25.19 Hz; and object 2 with spectral content of 1500 Hz and temporal content of 12.29 Hz. The stimulus duration was 1 s with an ISI and a delay period of the same length. Auditory stimuli were generated online at a sampling rate of 44.1 kHz and 16-bit resolution in Matlab 7.12.0 (Mathworks Inc.) and presented using Cogent (http://www.vislab.ucl.ac.uk). Sounds were played at participants' desired intensity, which was within the range of: 80–90 dB rms. Sounds were delivered diotically over headphones (Sennheiser HD 650) in a soundproof testing room.

### Design and procedure

**Figure 1:**
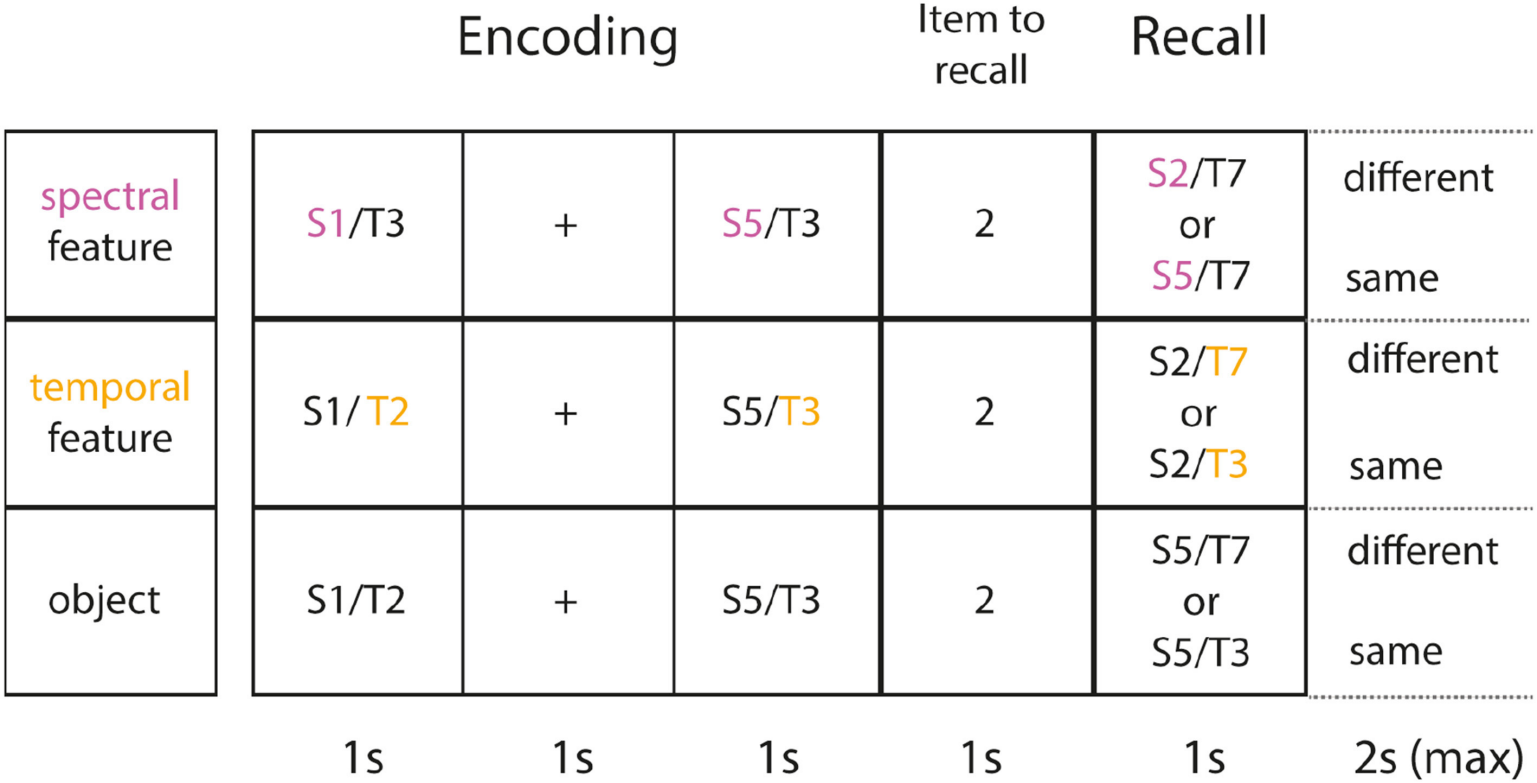
**Task and experimental conditions**. Shown are sample trials for each experimental condition (each row illustrates one of 3 conditions). Note that the same material (2 auditory objects) is presented at encoding (identical across conditions). Each object is presented for 1 s followed by an ISI of 1 s. Next, a number appears onscreen for 1 s, indicating which item in the sequence gets probed (here: 2 for 2nd item in the sequence). A final object is then presented and subjects have a maximum of 2 s to decide, whether the object or feature of interest is the same or different from the item tested (here: 2nd item). In the spectral condition (1st row) subjects only focus on the spectral feature (in purple). In the temporal condition (2nd row) subjects only focus on the temporal feature (in yellow). In object condition, they encode the object as a whole (both features in combination).

On each trial, participants listened to a sequence of sounds of variable length: 1, 2, or 4 auditory objects; see Figure 1. At the end of each sequence recall for one of the objects was probed, indicated by a number appearing onscreen, e.g., 2 for second sound. Each sound within the sequence was equally likely to be probed. A final auditory object was presented as the probe sound and subjects had to decide whether it was the same or different from the target sound (second sound, in Figure 1). They responded by pressing a button for “same” or “different.” There was a time window of 2 s to make a response. Memory performance was measured as accuracy of recall (percentage correct).

Further details on the selection of sound features at encoding and recall are given. On each trial, each auditory object was composed of two unique sound features, indicating that if a sequence contained more than a single item the same spectral or temporal features could not be repeated. To illustrate this further, a possible sequence for two objects was: S1/T3, S5/T2, whereas: S1/T3, S1/T2 was not possible. The minimum distance between any two temporal stimuli was 28% and the maximum distance was 196%. The minimum distance between any two spectral stimuli was 29% and the maximum distance was 203%. The distance between any two objects composed of each dimension was selected at random. Similar applies to the size of change between target and probe. There was no difference in task performance for different sizes of change between target and probe stimuli across conditions and memory loads.

Participants started the experiment by completing a practice block of 48 trials. Once they were familiarized with the sound attributes as well as the different experimental conditions, they completed the main experiment, consisting of 9 blocks of the same length with equal number of trials for each memory load. There were three types of experimental conditions (3 × 3 blocks), which took approximately 90 min to complete. In a second session, subjects completed 6 blocks of two types of control conditions (2 × 3 blocks), which could be completed in 60 min. An additional control condition was added later on, which was tested on a new set of participants consisting of 3 blocks (duration: 30 min).

#### Experimental conditions

There were two conditions in which subjects had to memorize single attributes of auditory objects and another condition in which they memorized objects as a whole (see Figure 1 for an overview of all conditions). In *the spectral condition*, they had to focus on the spectral content of the objects whilst ignoring the other attribute (temporal AM rate). Their memory for the spectral content was tested by 2AFC. On “same” trials, the amplitude modulation could be different, while the spectral content was identical to the target. On “different” trials, there was a change on both features.

In *the temporal condition*, subjects focussed on the AM rate (temporal content), whilst ignoring the other attribute (spectral content). Their memory for the attended attribute was then tested. In *the object condition*, subjects memorized the objects as a whole, forming bound percepts of both attributes. They were probed at random on either dimension. On “same” trials there was no change, whilst on “different” trials both features contained in the object changed.

It is important to note that stimulus presentation and probe selection were identical among all experimental conditions. A particular ink color of text appearing onscreen was assigned to each condition (spectral condition = pink, temporal condition = green, object condition = white). Experimental blocks were randomly interleaved and subjects knew by the ink color of text appearing onscreen which condition they were on.

#### Control conditions

**Figure 2:**
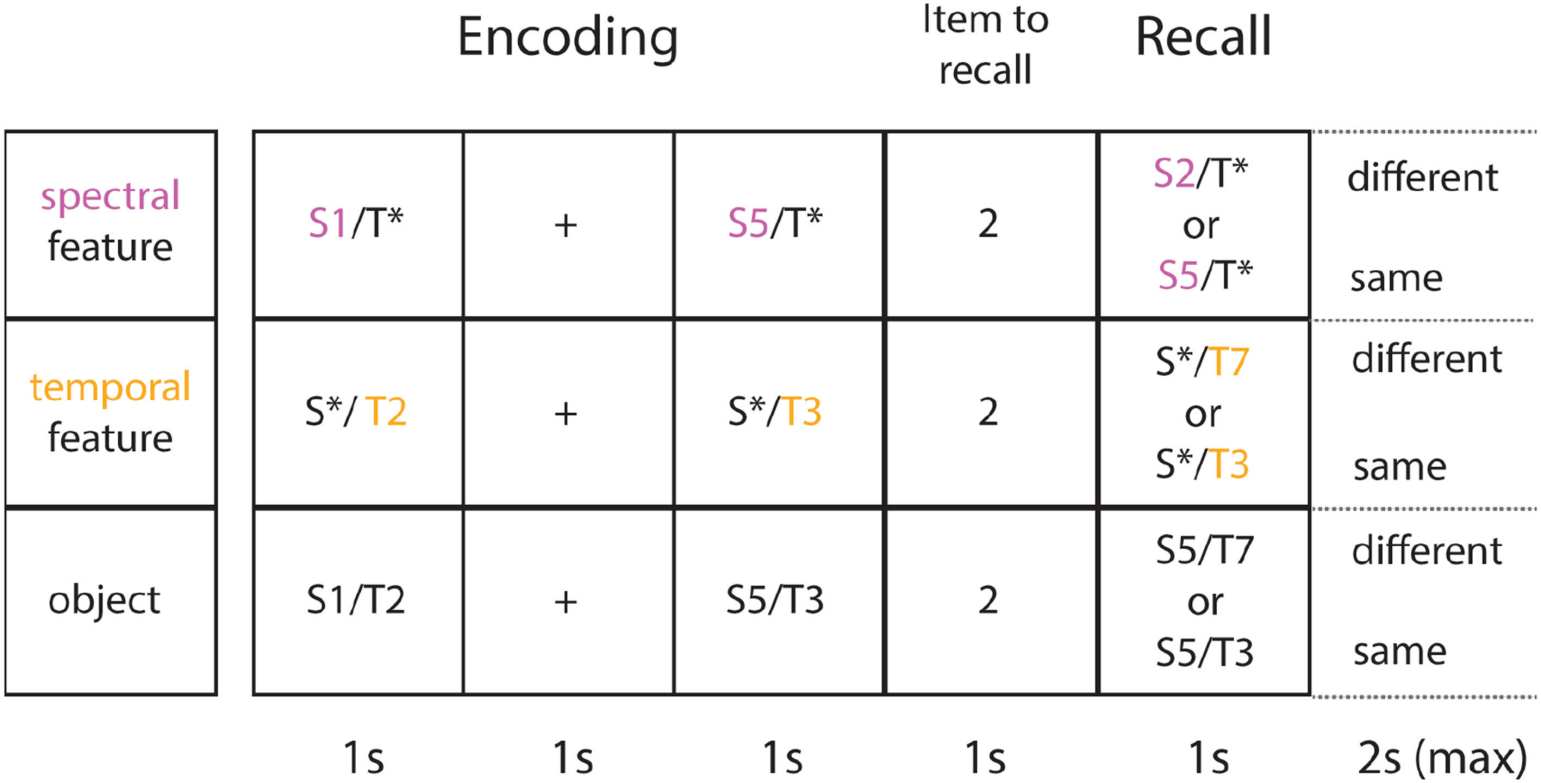
**Task and control conditions**. Shown are sample trials for each control condition (each row illustrates one of 3 conditions). In the spectral control condition (1st row) subjects focus only on the spectral feature (in purple). In the temporal control condition (2nd row) subjects only focus on the temporal feature (in yellow). In the object control condition (3rd row), they encode the object as a whole (both features in combination). Both single feature control conditions differ from the single feature experimental conditions in the following way: the irrelevant feature (*) is held constant at the middle value of the corresponding stimulus range. The object control condition differs from the experimental object condition in the way that on change trials, the item presented at recall (probe) differs from the target (here: 2nd item in the sequence presented at encoding) by 1 feature, instead of 2 features.

There was a control condition (see Figure 2) for each of the three experimental conditions. When a single feature (e.g., spectral) had to be maintained in WM, the other irrelevant dimension (e.g., temporal) may have caused interference with the relevant dimension of sound. In the experimental condition the dimension of the irrelevant sound feature was varied at random. However, the irrelevant dimension was held constant in the control condition in order to capture the amount of interference when comparing across conditions (experimental vs. control).

The *spectral control condition* was identical to the experimental spectral condition, where subjects had to focus on the spectral content of each object whilst ignoring the other attribute (temporal AM rate). However, the amplitude modulation rate was not selected at random as in the experimental condition, but held constant at 14 Hz (middle value of the temporal stimulus range). The *temporal control condition* was identical to the experimental temporal condition, where subjects focused on the AM rate, while ignoring the spectral content, which was held constant at 600 Hz (middle value of the spectral stimulus range).

Importantly, the *object control condition* was identical to the experimental object condition: subjects memorized auditory objects as a whole. However, on different trials the probe only changed on a single feature instead of a perceptually larger change of 2 features on the experimental object condition. The object control condition was added to ensure that potentially better memory performance on the experimental object compared to the experimental single-feature conditions is not solely due to a perceptually larger change between the item to recall and item presented at test.

### Data analysis

Hypotheses regarding the effects of memory load and condition, on memory performance (accuracy) were tested by ANOVAs and LSD *post hoc t*-tests, as specified in the results.

## Results

### Effects of memory load and condition on memory performance

Memory performance was measured as accuracy (percentage correct) for each sequence length (memory load: 1, 2, or 4 sounds), all experimental and control conditions within each sequence.

**Figure 3:**
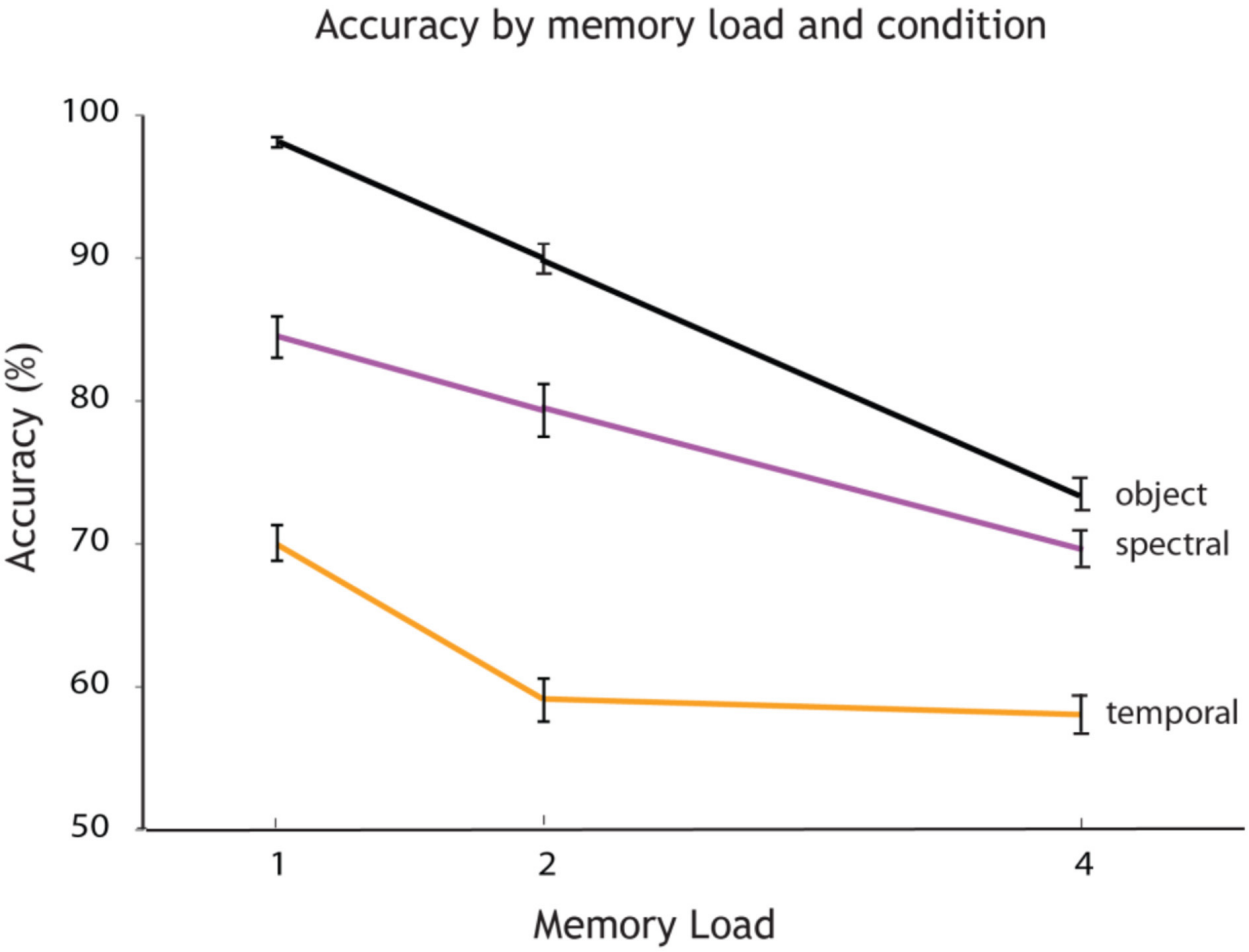
**Accuracy varies by memory load and experimental condition**. Overall accuracy (percentage correct) for every memory load (1, 2, and 4 auditory objects presented within a sequence). The plot shows how accuracy decreases with an increase in memory load for each experimental condition: single feature spectral condition (in rose), single feature temporal condition (in orange) and object condition (in black).

A Two-Way ANOVA was employed to test for the effects of factor 1, memory load, and factor 2, experimental condition, on accuracy. This analysis revealed a significant main effect of memory load [*F*_(2, 32)_ = 31.94, *p* < 0.001] and a significant main effect of experimental condition on accuracy [*F*_(2, 32)_ = 67.13, *p* < 0.001], as well as an interaction [*F*_(4, 32)_ = 2.72, *p* = 0.035], (see Figure 3). All *post hoc* comparisons between individual memory loads across conditions were significant at *p* < 0.001. Memory recall was more accurate when the object had to be maintained as a whole compared to its individual features. This result cannot be explained based on the level of difficulty: it would be expected that monitoring a single feature should be easier than two. Features therefore may be represented as bound units. Participants had more accurate recollection on the dimension of the spectral passband compared to the temporal AM rate.

**Figure 4:**
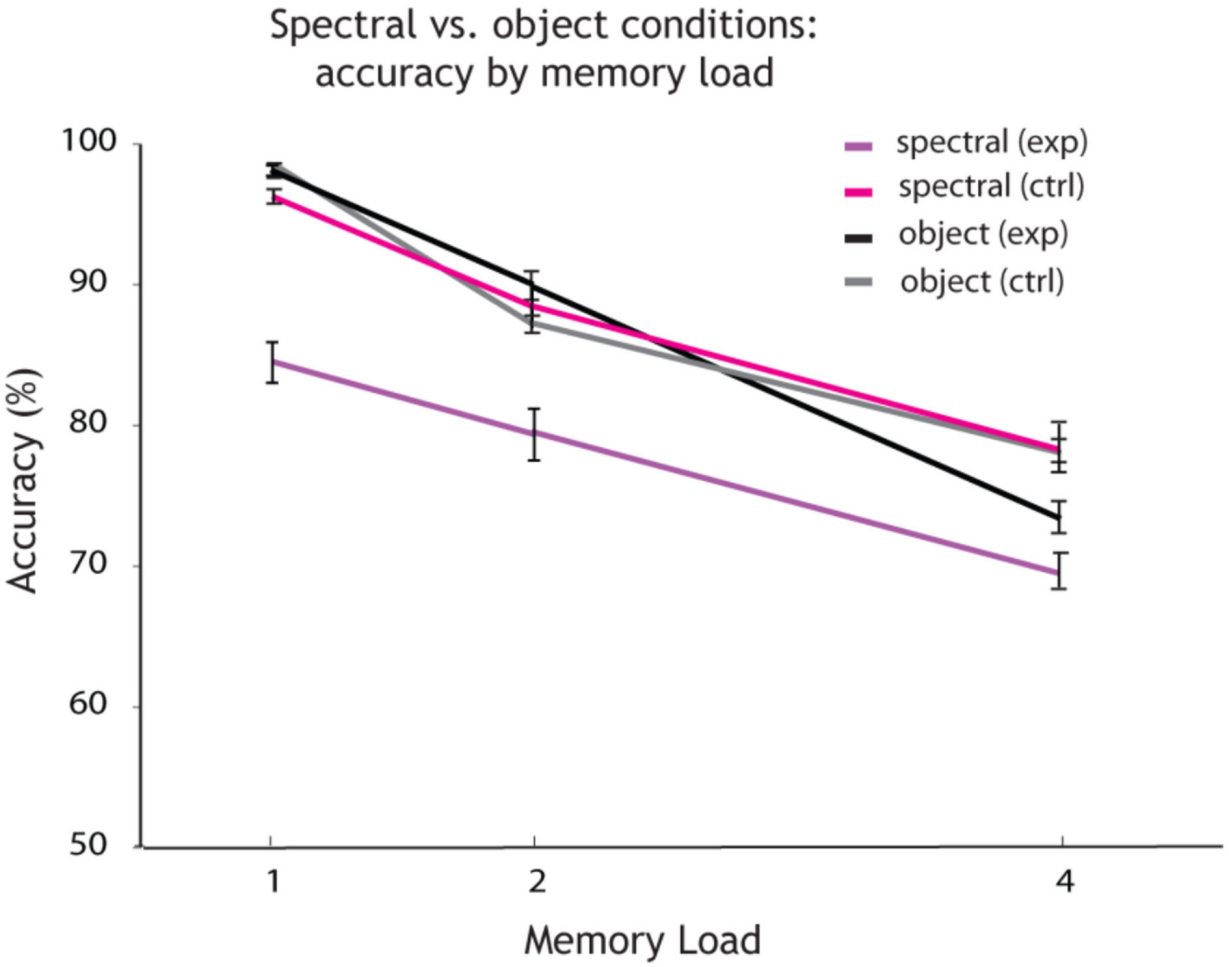
**Accuracy by memory load for single feature spectral vs. object conditions**. Overall accuracy (percentage correct) for every memory load (1, 2, and 4 auditory objects presented within a sequence). The plot shows how accuracy decreases with an increase in memory load for each condition. Shown in purple are the results for the single feature *spectral* condition. *Spectral* is the relevant and *temporal* the irrelevant feature varied at random. In pink: again *spectral* is the relevant feature, but the irrelevant *temporal* feature is held constant (spectral control condition). The results for the object condition are shown in black (the probe changes on both features on change trials). The results for the object control condition (the probe changes by a single feature on change trials) are shown in gray.

Further Two-Way-ANOVAs were carried out to compare the effects of memory load on accuracy across experimental and control conditions. Comparing the spectral condition (see Figure 4; purple) with the spectral control condition (pink), a Two-Way ANOVA revealed a significant main effect of memory load [*F*_(2, 32)_ = 47.8, *p* < 0.001] and a significant main effect of condition on accuracy [*F*_(1, 32)_ = 37.85, *p* < 0.001], as well as an interaction [*F*_(3, 32)_ = 5.46, *p* = 0.007], (see Figure 4). This comparison shows that varying the irrelevant feature at random (here: the temporal dimension of sound) leads to a decline in memory performance when tested on the spectral dimension of sound. Thus, the irrelevant feature interferes with encoding and recall of the relevant dimension of sound. Additionally a feature extraction cost is induced: the spectral dimension of sound is extracted from the object, while the temporal dimension induces a cost when varied at random. It is possible that a combination of both processes interference and feature extraction is at hand. However, we are unable to provide further evidence on feature extraction in relation to the time it took participants to respond, as reaction time data was not saved correctly.

**Figure 5:**
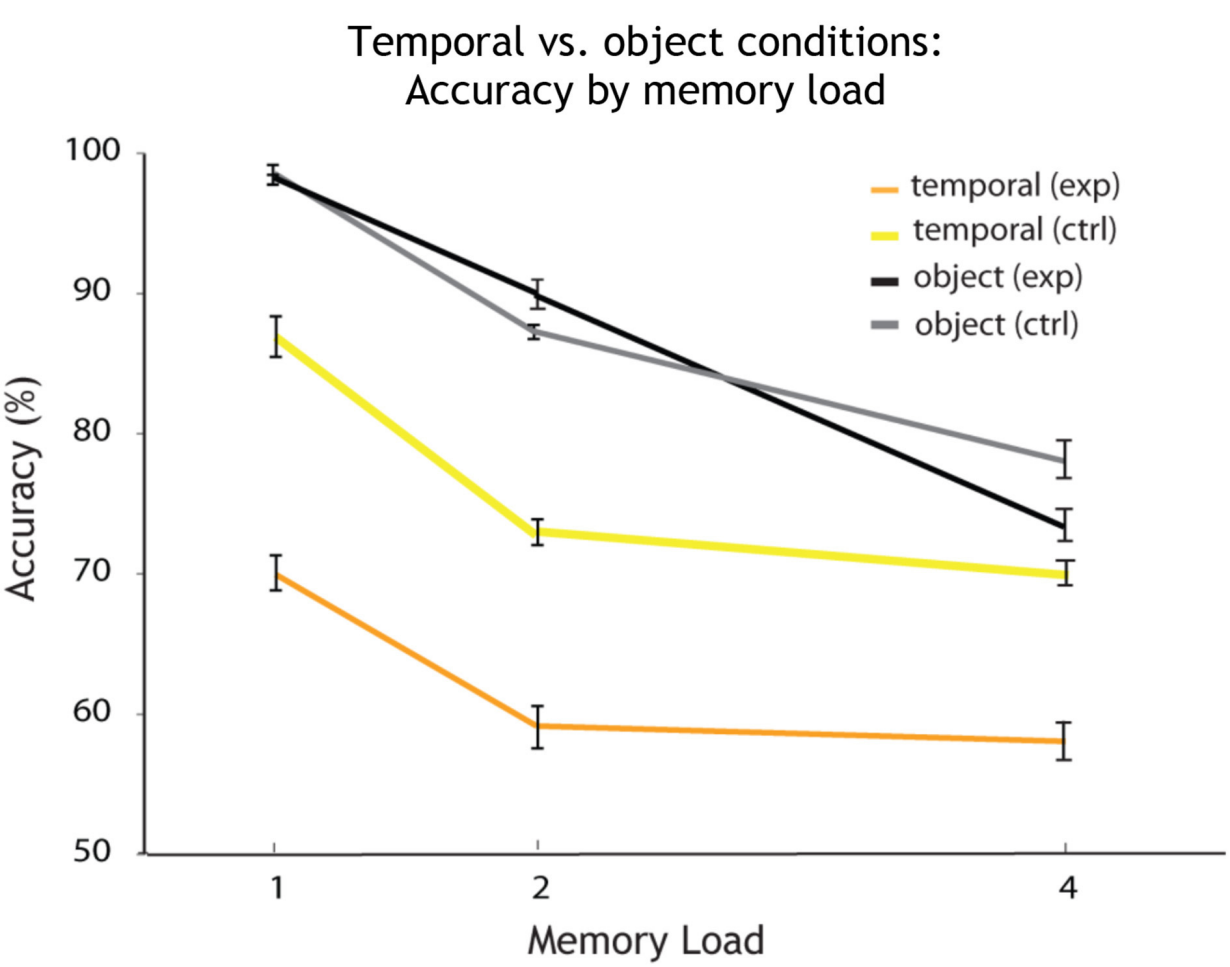
**Accuracy by memory load for single feature temporal vs. object conditions**. Overall accuracy (percentage correct) for every memory load (1, 2, and 4 auditory objects presented within a sequence). The plot shows how accuracy decreases with an increase in memory load for each condition. Shown in orange are the results for the single feature *temporal* condition. Temporal is the relevant and *spectral* the irrelevant feature varied at random. In yellow: again *temporal* is the relevant feature, but the irrelevant *spectral* feature is held constant (temporal control condition). The results for the object condition are shown in black (the probe changes on both features on change trials). The results for the object control condition (the probe changes by a single feature on change trials) are shown in gray.

Comparing the temporal condition (see Figure 5, in yellow) with the temporal control condition (in orange), a Two-Way ANOVA revealed a significant main effect of memory load [*F*_(2, 32)_ = 18.46, *p* < 0.001] and a significant main effect of condition on accuracy [*F*_(1, 32)_ = 44.97, *p* < 0.001], but no interaction (see Figure 5). This comparison shows that varying the irrelevant feature at random (here: the spectral dimension of sound) leads to a decline in memory performance when tested on the temporal dimension of sound. The irrelevant dimension of sound interferes with the relevant temporal dimension. Furthermore, extraction of the temporal dimension from the object comes at a cost, induced by the other dimension (spectral) when varied at random.

Further comparisons were carried out between the object condition (Figures 4, 5: in black) and the spectral control condition (Figure 4: in pink). A Two-Way ANOVA revealed a significant main effect of memory load on accuracy [*F*_(1, 32)_ = 49.47, *p* < 0.001], but no main effect of condition and no interaction. There was no difference in memory performance when the object was maintained as a whole compared to maintaining only its spectral content (single feature), while the irrelevant dimension (temporal) is held constant.

Next, the object condition (Figures 4, 5: in black) was compared with the temporal control condition (Figure 5: in yellow). A Two-Way ANOVA revealed a significant main effect of memory load [*F*_(2, 32)_ = 47.8, *p* < 0.001] and a significant main effect of condition on accuracy [*F*_(2, 32)_ = 37.85, *p* < 0.001], as well as an interaction [*F*_(4, 32)_ = 5.46, *p* = 0.007]. There was a difference in memory performance when the object was maintained as a whole compared to maintaining only its temporal content (single feature), while the irrelevant dimension (spectral) is held constant.

A final comparison was made between the object condition (Figures 4, 5: in black) and its corresponding object control condition (Figures 4, 5: in gray). A Two-Way-ANOVA revealed a main effect of memory load [*F*_(2, 20)_ = 65.8, *p* < 0.001], but no effect of condition [*F*_(1, 20)_ = 0.176, *p* = 0.667] and no interaction [*F*_(3, 20)_ = 1.85, *p* = 0.166]. The size of change between the item tested and the item presented at recall (by 1 or 2 features) did not make difference in memory performance, when maintaining objects as a whole.

## Discussion

We investigated the basis for holding auditory stimuli in WM by analysing listeners' memory performance when they either maintained objects as a whole or their component spectral and temporal features. Memory recall was more accurate when the object had to be maintained as a whole compared to retaining individual features. One interpretation of these findings is that at some level of processing sounds are stored as objects in WM: (1) due to interference of features within objects; (2) a feature extraction cost is induced, when storing individual features in WM. This shows that we naturally remember sounds as bound objects even when asked to only memorize one of their component features. Thus, such feature binding might serve as a mechanism to increase WM capacity. We do not dismiss the existence of distinct mappings of temporal and spectral stimulus properties in the auditory system, for which there is good evidence in the ascending auditory system (Baumann et al., 2011) and cortex (Barton et al., 2012). However, the present data support such stimulus properties being combined into objects before encoding into working memory. A number of previous studies reviewed in Griffiths and Warren (2004), Bizley and Cohen (2013) support the existence of auditory objects as coherent units of perception in which acoustic components are grouped by cues such as common onset, harmonicity and common modulation. A further possible interpretation of our data is that such perceptual units are the input stage for auditory working memory.

### Holding whole objects in mind

In our data, at the object level, performance was at ceiling for the memory load of a single item and then dropped with the addition of each further item to be held in working memory. Performance remained above chance (>50% accuracy) at the highest memory load of four auditory objects. A similar pattern was observed in both object conditions and was independent of the size of change of the probe stimulus (on different trials) relative to the target stimulus—by two features (object condition) or by one feature (object control condition, see Figure 4). The size of change of the probe stimulus did not lead to a difference in performance across memory loads. These results support the idea that at some level of processing information retrieved from working memory at the object level is not sensitive to the number of feature dimensions that change (i.e., a change of one or two dimensions).

### Holding individual features in mind: spectral passband

When only the dimension of spectral passband was held in mind, whilst the other ignored dimension was held constant (*spectral control condition*), memory performance was equal to performance in the object condition. Thus, for this particular dimension, holding an object or a feature in mind draws equally on memory resources. Here, WM appears to be object based (in line with Mathias and Von Kriegstein, 2014). We naturally remember sounds as bound objects, depending on how we define an auditory object. If an auditory object is primarily defined by a single dimension of sound (one feature), this feature can be regarded as an object, similar to an object composed of multiple features. Furthermore, comparison of memory performance across conditions in which only the dimension of spectral content had to be maintained (*spectral condition* where the other dimension was varied randomly, rather than held constant as in the spectral control condition), demonstrates better performance in the control compared to the experimental condition (see Figure 4). This difference in memory performance can be explained by an interference of the irrelevant with the relevant dimension (experimental condition), which is greatly reduced when the irrelevant dimension is held constant (control condition). Additionally, this comparison shows that there is a significant cost in feature extraction. We suggest that both processes interference and feature extraction may act in combination, distorting the trace of the to be held in mind relevant feature.

When the irrelevant dimension of sound was varied randomly, it interfered with encoding of the feature of interest in a way that does not occur when the irrelevant dimension was held constant. In audition, it has been suggested that object formation depends on attentional processes (Shinn-Cunningham, 2008). Relevant to perception and memory encoding, previous research has shown that we can focus on a desired feature presented as part of an auditory object (Brungart and Simpson, 2002; Rakerd et al., 2006; Shinn-Cunningham, 2008; Helfer and Freyman, 2009) and that task irrelevant features influence selective attention (Maddox and Shinn-Cunningham, 2012). Thus, some of the observed cost in feature extraction may arise at the stage of memory encoding, as attention has to be allocated to extract the feature of interest. As a result, the interference caused by the irrelevant dimension may add noise to the memory representation of the feature of interest in WM. The representation's saliency is reduced by partial feature overwriting, as has previously been demonstrated for dimensions of timbre (Mercer and McKeown, 2010).

However, at the stage of memory retrieval, when deciding whether the probe matches the target stimulus, one has to focus on the probe's relevant feature dimension while ignoring the other dimension. The relative cost of extraction of the relevant dimension of sound is larger when the irrelevant dimension is varied randomly compared to when it is held constant (spectral control condition). Therefore, at the stage of memory retrieval, the irrelevant dimension of the probe may also worsen WM performance. In sum, the observed cost in feature extraction may be due to several sources of interference related to the irrelevant dimension at different stages of WM (encoding, maintenance and recall). Future research using neuroimaging methods (e.g., event-related potentials with a high temporal resolution), might potentially be able to disentangle the time-course of events of the different stages of memory processes involved to clarify information when there is loss in WM representations of features vs. objects. Additionally, future research shall determine the extent to which information loss is due to interference between features in contrast to the cost induced by feature extraction.

### Holding individual features in mind: temporal content

On the individual feature level, with regards to the temporal AM rate, performance was lower when the other dimension (now spectral) was held constant (*spectral control condition*) compared to the object condition. This is unlike the comparison between the corresponding spectral conditions (spectral control condition vs. object condition), where performance was equal across conditions. In the single-feature experimental conditions, although the spacing of stimuli along each feature dimension was based on perceptual thresholds, it is possible that they might not have been perfectly matched in terms of difficulty. Thus, the observed difference across conditions (spectral vs. temporal) might reflect that it was simply easier to hold spectral features in mind compared to temporal ones.

If the level of difficulty was matched across conditions, a different interpretation is that the spectral passband (including perceived variations in loudness) may represent a stronger cue for object formation. In other words, the spectral dimension of sound defines the object to a larger extent compared to its temporal dimension. It may dominate object formation at encoding or override temporal representations at maintenance. Alternatively, WM stores for either feature dimensions may be partly independent, where each store has a different capacity limit. In this case, the capacity limit for the temporal dimension would be lower compared to the capacity limit found on the other dimension. However, it would be important to reproduce the findings presented here with different stimulus spacing to verify such a possibility.

Additionally, we considered indirect mechanisms for the interaction between frequency and modulation rate via other sound dimensions including loudness. Whilst the modulation depth and therefore rms level were fixed the change in frequency will have produced a small change in perceived loudness, which we estimate to be 2–5% based on ISO equal loudness curves. It is theoretically possible that loudness contributed to the interaction between AM rate and center frequency but working memory for loudness is poor over durations of more than 2 s and likely uses a distinct mechanism to that for pitch (Clément et al., 1999).

Moreover, comparing memory performance across conditions on which temporal information has to be maintained (*temporal condition* where the other dimension was varied randomly vs. *temporal control condition* where it was held constant), performance was better on the control compared to the experimental condition (see Figure 5). Similar to the results already discussed for spectral passband, this difference in memory performance can be explained by an interference of the irrelevant with the relevant dimension (experimental condition). Thus, performance improves when the irrelevant dimension is held constant (control condition). Additionally, there is a significant cost in feature extraction on the temporal dimension, similar to our findings for the spectral dimension (spectral condition vs. spectral control condition).

The observed cost in feature extraction on the temporal dimension of sound may also be based on several different sources of interference caused by the irrelevant dimension (spectral) at different stages of WM (encoding, maintenance and retrieval). The extent of this interference seems to be larger when comparing across temporal conditions (temporal condition vs. temporal control) compared to spectral conditions (spectral condition vs. spectral control), as the drop in memory accuracy is more severe when extracting the temporal feature, which may again be due to the design of the temporal stimulus dimension (not necessarily being equated in terms of difficulty to the spectral dimension). Alternatively, this asymmetry in interference might also be due to uneven amounts of interference from the irrelevant feature dimension. For example, when the spectral dimension was varied randomly (irrelevant dimension), it interfered more with the relevant feature (temporal), than the interference caused by the temporal dimension (irrelevant) on the spectral dimension (relevant).

### Relation to feature integration theory

Object formation involves binding of features, which become reorganized to create more complex unified representations of previously distributed information (Treisman and Schmidt, 1982). A previous study of auditory objects composed of either 3 or 6 dimensions of timbre, showed that WM capacity increased, when the acoustic separation between the probe and test items increased, as well as the number of feature dimensions (Golubock and Janata, 2013). In relation to feature integration theory and in particular feature overwriting (distinct neural populations code for different dimensions of timbre), they showed that capacity is facilitated when stimuli have un-shared features. Thus, there is less competition between any two features for “representational space” in corresponding neural regions. Our results are difficult to directly compare to this study, as our auditory objects were always composed of the same two dimensions, where not the number, but relevance of features was manipulated. Apart from a single previous and the current study, little is known about the organization of auditory object features in auditory WM.

However, another recent study has shown that the units of storage of auditory information are at the object level (Mathias and Von Kriegstein, 2014). The authors showed that information (distractors) presented in the delay period influenced recall of auditory objects. Participants had to hold a single object in mind, composed of three features: two spatial ones (interaural time difference and interaural level difference) as well as sound frequency. The features participants had to focus on were varied on a trial-to-trial basis. If they held a spatial feature in mind and the information presented in the delay period was varied along this dimension, there was greater interference on recall than when distractors were varied along the frequency dimension (and vice versa). Thus, the study mostly investigated how information from distractors influences recall of single auditory objects, but not how the different types of information contained within a single object influences each other. Apart from fundamental differences in the design of their study compared to the current experiment, our results are in line with the interpretation that at some level in the auditory system, information is represented, as objects in auditory WM. However, this view need not be mutually incompatible with the proposal that auditory stimuli might also be stored as features within some representations, echoing some of the debate that has occurred in the visual domain. Future research might profitably address the fidelity or precision of auditory object representations, which has already been described for sound frequency (Kumar et al., 2013). It would be interesting to measure recall precision across sound features to determine trade-off relationships between and across features of auditory objects held in WM.

## Conclusion

The results presented here show that once the information is unified into an auditory object, manipulating it at memory retrieval by extracting single features decreases memory accuracy. One interpretation of our findings is that, at some level, sounds may be stored as objects, as there is an extraction cost for single features stored in WM. However, information processing of features in WM may be more complex than this. Features are represented at different levels in the auditory systems and may only be bound and interfere with each other at some level in this hierarchy. The difference in memory performance across individual feature conditions may depend on the definition of each feature dimension (spacing of stimulus range) rather than on how memory resources are allocated differently to either feature. It remains to be tested whether introducing different stimulus spacing along either feature dimension has an influence on memory performance. In this way, it would be possible to determine fully, whether it is more resource demanding to hold one or the other feature in mind.

### Conflict of interest statement

The authors declare that the research was conducted in the absence of any commercial or financial relationships that could be construed as a potential conflict of interest.
